# Flow Cytometric Assessment of TRBC1 Expression for the Diagnosis of T‐Cell Lymphoma: Clinical Utility and Pitfalls Related to T‐Cell Clones of Uncertain Significance

**DOI:** 10.1111/ijlh.14508

**Published:** 2025-06-16

**Authors:** SooHo Yu, Boram Kim, Sang Eun Yoon, Hee‐Jin Kim, Hyun‐Young Kim

**Affiliations:** ^1^ Department of Laboratory Medicine and Genetics Samsung Medical Center, Sungkyunkwan University School of Medicine Seoul Korea; ^2^ Division of Hematology and Oncology, Department of Medicine Samsung Medical Center, Sungkyunkwan University School of Medicine Seoul Korea

**Keywords:** flow cytometry, T‐cell clones of uncertain significance, T‐cell lymphomas, T‐cell receptor constant beta chain 1, TRBC1

## Abstract

**Background:**

T‐cell receptor constant beta chain 1 (TRBC1) has emerged as a potential clonal marker for T‐cell lymphoma. This study evaluated TRBC1 expression using flow cytometry in healthy individuals and various clinical specimens, assessing its clinical utility in the diagnosis of T‐cell lymphomas.

**Methods:**

Flow cytometric analysis was performed on peripheral blood specimens from 20 healthy individuals and 186 clinical specimens (116 bone marrow aspirates, 18 peripheral bloods, 52 body fluids) from 177 patients for the exclusion or differential diagnosis of T‐cell lymphoma using a panel of nine monoclonal antibodies: CD45, CD3, CD4, CD8, CD56, CD2, CD5, CD7, and TRBC1.

**Results:**

TRBC1 showed polytypic expression in healthy individuals with a median of 38.6% (range, 33.9%–46.1%) in CD4+ T cells and 26.3% (range, 10.9%–44.2%) in CD8+ T cells (*p <* 0.001). TRBC1 expression in non‐T‐cell lymphoma specimens was comparable to that in healthy peripheral blood, except for bone marrow, which showed slightly higher TRBC1 expression. All 20 T‐cell lymphoma cases exhibited monotypic TRBC1 expression, with a predominance of TRBC1− (65%). Additionally, T‐cell clones of uncertain significance (TCUS) were detected in 12.7% of non‐T‐cell lymphoma specimens, predominantly in CD8+ T cells and associated with cytopenic and reactive conditions.

**Conclusions:**

This study demonstrates the clinical utility of flow cytometric TRBC1 assessment in diagnosing T‐cell lymphomas, characterizing TRBC1 expression patterns, and identifying TCUS in a large cohort of specimens without T‐cell lymphoma involvement across diverse clinical settings. Given the relatively frequent detection of TCUS, these findings underscore the importance of interpreting TRBC1 flow cytometry results within the appropriate clinical context.

## 1 Introduction

T‐cell lymphomas are a heterogeneous group of hematologic malignancies with various presentations and molecular complexities, making them diagnostic challenges [1]. Flow cytometry has played an important role in identifying clonal T‐cell populations for T‐cell lymphoma diagnosis. As a single‐cell‐based method, flow cytometry offers the advantage of simultaneously evaluating clonality and immunophenotype in both total T‐cell populations and T‐cell subsets. However, unlike B‐cell lymphomas, evaluating T‐cell lymphoma remains challenging due to the lack of definitive clonal marker in flow cytometry [2]. While TCRVβ repertoire analysis by flow cytometry is available, its clinical application is limited by the requirement of numerous antibodies [3, 4]. Antigen loss in reactive conditions also poses a diagnostic challenge in differentiating between reactive conditions and T‐cell lymphomas [5].

Recently, a monoclonal antibody specific for human T‐cell receptor (TCR) constant beta chain 1 (TRBC1) has been introduced as a potential clonal marker in αβ T‐cell lymphomas, based on the mutually exclusive expression of constant regions C1 and C2 of the β chain during αβ TCR rearrangement [6]. Labeling T‐cell populations with TRBC1‐ or TRBC2‐specific antibodies produces distinct positive or negative signals, similar to using kappa and lambda light chain antibodies in B‐cell lymphomas [7, 8]. A TRBC1‐specific monoclonal antibody was initially commercialized, with a TRBC2‐specific monoclonal antibody recently introduced.

In this study, we evaluated TRBC1 expression in healthy individuals and various clinical specimens, and assessed its clinical utility in patients with suspected T‐cell lymphoma using flow cytometry.

## 2 Materials and Methods

### 2.1 Study Subjects

This study included patients who underwent flow cytometric T‐cell analysis at Samsung Medical Center between March and December 2023. Flow cytometric analysis was performed on peripheral blood specimens from 20 healthy individuals and 186 clinical specimens from 177 patients for the exclusion or differential diagnosis of T‐cell lymphoma. Clinical specimens consisted of three types of specimens: bone marrow aspirate (*n* = 116), peripheral blood (*n* = 18), and body fluids (*n* = 52) including cerebrospinal, pleural, ascitic, and pericardial fluids. T‐cell lymphomas were diagnosed according to the fifth edition of the WHO classification of Haematolymphoid Tumours [9]. T‐cell clones of uncertain significance (TCUS) were defined as small populations of clonal T cells exhibiting monotypic TRBC1 expression on immunophenotypically aberrant T cells, likely representing dominant T immunoclones [6, 10]. Patients' laboratory data, including complete blood count, bone marrow and/or tissue biopsy results, flow cytometry results, and/or circulating Epstein–Barr virus (EBV) DNA, as well as clinical information, were obtained from electronic medical records. This study was approved by the Institutional Review Board of Samsung Medical Center, Seoul, Korea (2024‐09‐004), with a waiver of informed consent due to its retrospective design.

### 2.2 Flow Cytometric T‐Cell Analysis

Flow cytometry analysis was performed as previously described with the addition of an anti‐TRBC1 antibody (clone JOVI‐1, Ancell Corporation, Bayport, Minnesota) [11]. The flow cytometric T‐cell analysis panel consisted of nine monoclonal antibodies in a single tube: CD45‐V500‐C (Becton, Dickinson and Company [BD], New Jersey, United States), CD3‐APC‐Cy7 (BD), CD4‐Pacific Blue (Beckman Coulter [BC], California, United States), CD8‐PE‐Cy7 (BD), CD56‐APC (BD), CD2‐PerCP (BD), CD5‐FITC (BD), CD7‐PE (BD), and TRBC1‐BV605. After surface staining, red blood cells were lysed using FACS Lysing Solution (BD). Flow cytometry was performed using a FACSLyric Flow Cytometer (BD), acquiring a total of 2 × 10^5^ events per sample. Data were analyzed with Kaluza analysis software (BC) as follows: debris was removed using a forward scatter (FSC)/side scatter (SSC) plot, then lymphocytes were gated on a CD45/SSC plot. CD3+ T cells were identified and subdivided into CD4+ and CD8+ T‐cell populations. Expression of CD2, CD5, CD7, and TRBC1 was assessed in both subpopulations. As TCRαβ and TCRγδ antibodies were not used, γδ T cells were excluded based on characteristic immunophenotypes (i.e., CD4−/CD8− or CD8dim+ patterns), and the remaining cells were considered αβ T cells for TRBC1 expression analysis. TRBC1 expression was evaluated in T‐cell populations with ≥ 50 events, and populations below this threshold were considered uninterpretable. Additionally, CD2, CD4, CD5, CD7, and CD8 expression was assessed in CD3− lymphocytes to confirm the presence of CD3− T cells. Clonal T‐cell populations were identified by examining aberrant expression (i.e., loss, ectopic expression, decreased intensity, and/or abnormally bright expression) of CD3, CD4, CD8, CD2, CD5, and/or CD7, along with monotypic expression of TRBC1. Based on previous studies [6, 12, 13, 14, 15], we arbitrarily defined clonal (monotypic) TRBC1 expression as TRBC1 expression either below 10% or above 90% in the target T‐cell population.

### 2.3 Statistical Analysis

All statistical analyses were performed using IBM SPSS Statistics, version 27 (IBM Corp., NY, USA) and R 4.4.1 software (Wien, Austria). The Shapiro–Wilk and Kolmogorov–Smirnov tests were performed to check the normality of the variables. The Mann–Whitney test or two‐sample *t*‐test was used to compare continuous variables, as appropriate. Values are presented as mean with 95% confidence interval (CI) or median with range, depending on the distribution of data. *p* < 0.05 was considered statistically significant.

## 3 Results

### 3.1 TRBC1 Expression in Healthy Specimens

First, TRBC1 expression was analyzed in peripheral blood from healthy individuals (*n* = 20; male:female ratio 1:1; median age 61 years [range, 46–79 years]). All subjects showed polytypic TRBC1 expression, with no significant differences between males and females (*p* > 0.05). The median TRBC1 expression was significantly higher in CD4+ T cells at 38.6% (range, 33.9%–46.1%) compared to CD8+ T cells at 26.3% (range, 10.9%–44.2%) (*p <* 0.001) (Table 1). CD8+ T cells demonstrated greater variability with a standard deviation of 9.0% compared to 4.1% for CD4+ T cells. The 95% reference intervals for TRBC1 expression were 31.4%–47.8% for CD4+ T cells and 8.7%–44.7% for CD8+ T cells, with median TRBC1+/TRBC1− ratios of 0.61 (range, 0.50–0.82) and 0.35 (0.12–1.12), respectively (*p <* 0.001).

**TABLE 1:** TRBC1 expression in peripheral blood specimens from healthy individuals and various clinical specimens without T‐cell lymphoma involvement.

Specimen	Healthy specimens	Non‐T‐cell lymphoma specimens		
	PB (*n* = 20)	PB (*n* = 8)	BM (*n* = 109)	BF (*n* = 49)
CD4+ T cells				
Median TRBC1 expression	38.6%	40.4%	42.1%	41.2%
Range	33.9%–46.1%	21.3%–48.3%	23.4%–61.3%	18.6%–61.3%
CD8+ T cells				
Median TRBC1 expression	26.3%	32.7%	32.4%	31.5%
Range	10.9%–44.2%	16.6%–80.8%	10.1%–67.8%	14.3%–66.7%

Abbreviations: BF, body fluid; BM, bone marrow; PB, peripheral blood.

### 3.2 TRBC1 Expression in Non‐T‐Cell Lymphoma Specimens

**FIGURE 1:**
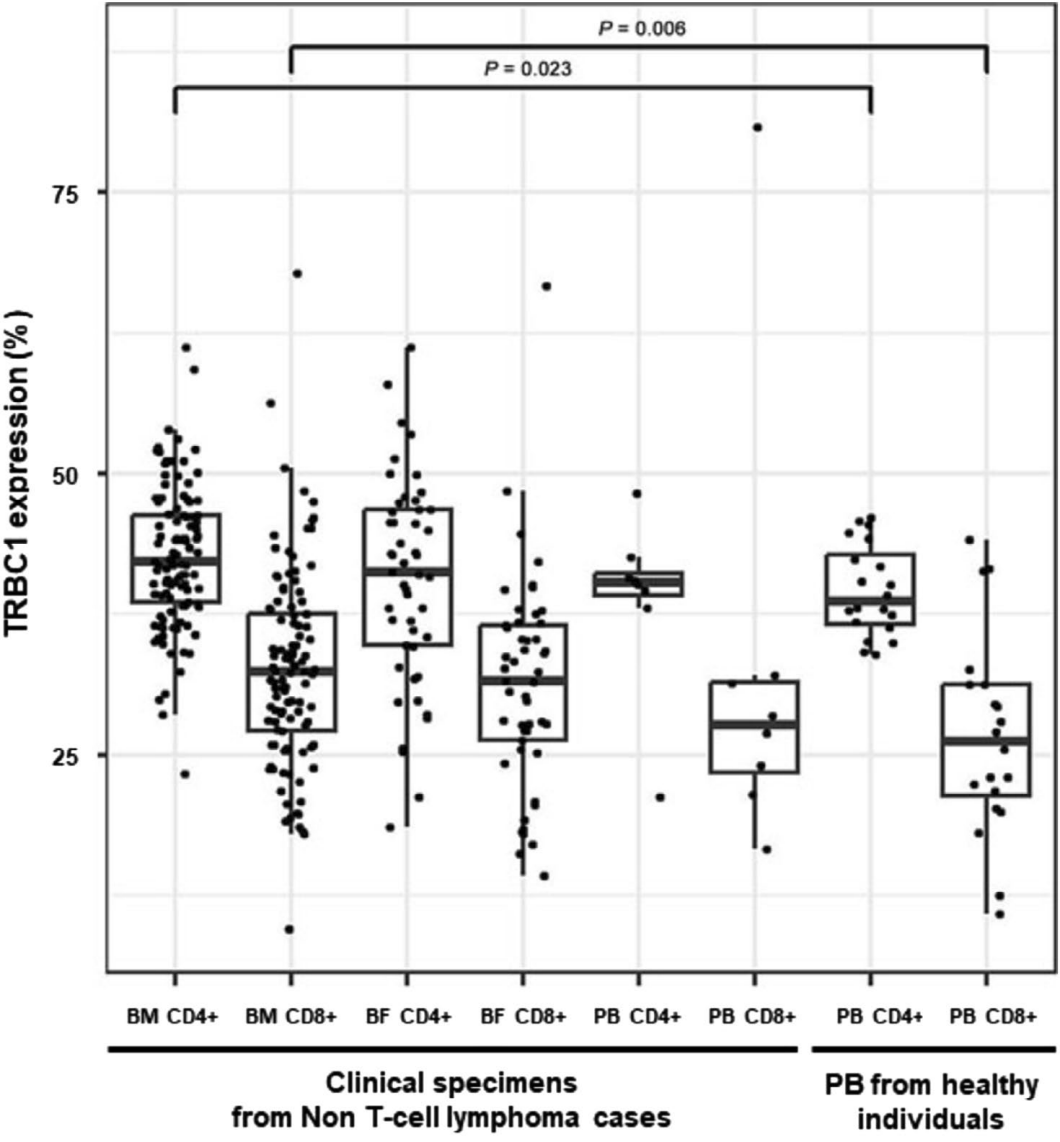
TRBC1 expression in CD4+ and CD8+ T cells from various clinical specimens in non‐T‐cell lymphoma cases and in peripheral blood (PB) of healthy individuals. TRBC1 expression was significantly higher in CD4+ T cells than in CD8+ T cells across all specimen types (all *p* < 0.001). Compared to PB from the healthy individuals, both CD4+ and CD8+ T cells from bone marrow (BM) showed significantly elevated TRBC1 expression (*p* = 0.023 and *p* = 0.006, respectively), whereas no significant differences were observed in T cells from body fluid (BF) and PB (all *p* > 0.05).

Next, we examined TRBC1 expression in non‐T‐cell lymphoma specimens (*n* = 166). The clinical information for these specimens is described in Table S1. The median TRBC1 expression in CD4+ T cells ranged from 40.4% to 42.1% across bone marrow aspirates, body fluids, and peripheral blood specimens, while CD8+ T cells ranged from 31.5% to 32.7%. Similar to healthy individuals, TRBC1 expression was significantly higher in CD4+ T cells compared to CD8+ T cells (all *p* < 0.001). No significant differences in TRBC1 expression were observed between specimen types in either CD4+ or CD8+ T cells (all *p >* 0.05) (Figure 1). However, both CD4+ and CD8+ T cells showed slightly higher TRBC1 expression in bone marrow compared to healthy peripheral blood (*p* = 0.023 and *p* = 0.006, respectively).

To further explore whether reactive conditions influenced TRBC1 expression, we analyzed TRBC1 in a subgroup of 32 bone marrow specimens with reactive changes. In these specimens, TRBC1 expression in CD4+ T cells ranged from 23.36% to 52.36% (median 41.46%), and in CD8+ T cells from 20.68% to 47.49% (median 32.23%). These values were comparable to those observed in the overall non‐T‐cell lymphoma bone marrow specimens (all *p* > 0.05).

We also investigated TRBC1 expression in various minor T‐cell subpopulations in bone marrow aspirates, regardless of the number of gated events. In CD3+CD4+CD8+ T cells, TRBC1 expression ranged from 0% to 81.3% (median 40.4%). In CD3+CD4−CD8− T cells, presumed to be primarily γδ T cells, TRBC1 expression ranged from 0% to 51.9% (median 7.7%). Additionally, TRBC1 expression was assessed in commonly observed T‐cell subpopulations, independent of CD4 or CD8 expression, including CD3+CD7−, CD3+CD7+CD5−, and CD3+CD7+CD5+CD2− T cells. The TRBC1 expression ranges for these subpopulations were 8.5%–76.5% (median 44.0%), 6.9%–86.1% (median 28.5%), and 0%–90.9% (median 45.0%), respectively. Notably, skewed TRBC1 expression levels (below or above 10%) were often seen in these minor subpopulations, typically attributed to a small number of gated events (< 50 events).

### 3.3 TRBC1 Expression in T‐Cell Lymphomas

**FIGURE 2:**
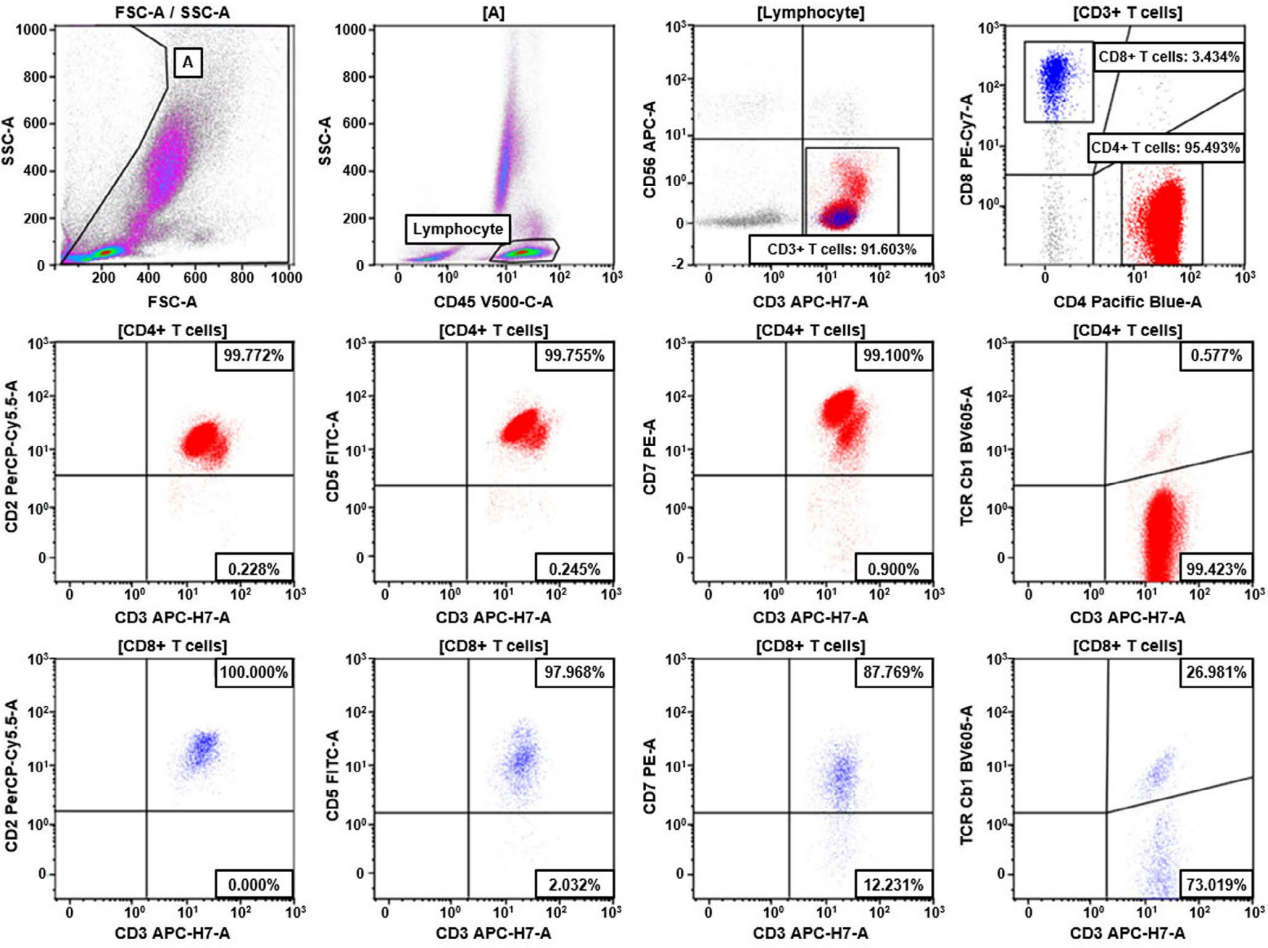
Flow cytometric analysis of TRBC1 expression in a patient (T‐9) with T‐cell prolymphocytic leukemia. CD4+ T cells (red dots) show marked increase and demonstrate monotypic loss of TRBC1 expression (TRBC1–), indicating clonal expansion. In contrast, CD8+ T cells (blue dots) exhibit polytypic TRBC1 expression. Neither subset demonstrates aberrant expression of CD2, CD5, or CD7.

Twenty specimens (seven bone marrow aspirates, three body fluids, and 10 peripheral blood specimen) from 18 patients were diagnosed with mature T‐cell lymphoma infiltration histologically (anaplastic large‐cell lymphoma, *n* = 3; extranodal NK/T‐cell lymphoma, *n* = 1; peripheral T‐cell lymphoma, *n* = 2, systemic EBV+ T‐cell lymphoma, *n* = 1; T‐cell prolymphocytic leukemia, *n* = 2; T‐cell lymphoma, NOS, *n* = 2; follicular helper T‐cell lymphoma, *n* = 5; and T‐cell large granular lymphocytic leukemia [T‐LGL], *n* = 4) (Table 2). In two patients, T‐cell lymphoma infiltration was identified in two distinctive specimens—peripheral blood (T‐16) and bone marrow (T‐14) in one patient, and cerebrospinal fluid (T‐3) and bone marrow (T‐2) in the other—with each specimen considered a separate case in this study. All cases showed monotypic TRBC1 expression in the target T‐cell population: TRBC1+ (30%, *n* = 6), TRBC1− (65%, *n* = 13), and homogeneously diminished TRBC1 expression (dim+) (5%, *n* = 1). Clonal T‐cell populations ranged from 0.45% to 92.20%. Sixteen cases (80%) exhibited aberrant CD2, CD5, or CD7 expression in conjunction with monotypic TRBC1 expression. The remaining cases (20%) were diagnosed based on monotypic TRBC1 expression in the majority of CD4+ or CD8+ T cells without aberrant antigen expression. A representative plot is shown in Figure 2.

**TABLE 2:** Clinical and immunophenotypic characteristics of T‐cell lymphoma cases (*n* = 20).

Case no.	Diagnosis	Specimen	Immunophenotype^a^	Proportion (%)
T‐1	ALCL	PB	CD3+, CD4+, CD2+, CD5+, CD7+, TRBC1+	20.56
T‐2	ALCL	BM	CD3+, CD8+, CD2+, CD5+, TRBC1+	6.64
T‐3	ALCL	CSF	CD3+, CD4+, CD2+, TRBC1+	79.05
T‐4	ENKTCL	PB	CD3+, CD8+, CD56−, CD2+, CD5+, CD7+, TRBC1−	7.28
T‐5	PTCL, NOS	AF	CD3+, CD4+, CD5+, CD7+, TRBC1−	16.50
T‐6	PTCL, NOS	PF	CD3+, CD4+, CD5+, CD7+, TRBC1+	11.87
T‐7	sEBV + TCL	BM	CD3+, CD8+, CD2+, CD7+, TRBC1−	0.45
T‐8	T‐PLL	PB	CD3+, CD4+, CD2+, CD5+, CD7partial+, TRBC1+	92.20
T‐9	T‐PLL	BM	CD3+, CD4+, CD2+, CD5+, CD7+, TRBC1−	28.33
T‐10	TCL, NOS	BM	CD3+, CD4+, CD7+, TRBC1−	28.32
T‐11	TCL, NOS	PB	CD3+, CD4+, CD2+, CD5dim+, CD7dim+, TRBC1+	29.86
T‐12	TFH lymphoma, angioimmunoblastic‐type	BM	CD3+, CD4+, TRBC1dim+	5.05
T‐13	TFH lymphoma, angioimmunoblastic‐type	PB	CD3+, CD4+, CD2+, CD5dim+, TRBC1−	34.14
T‐14	TFH lymphoma, NOS	BM	CD3+, CD4+, CD2+, CD5+, TRBC1−	20.66
T‐15	TFH lymphoma, NOS	PB	CD3+, CD4+, CD2+, CD5+, TRBC1−	4.90
T‐16	TFH lymphoma, NOS	PB	CD3+, CD4+, CD2+, CD5+, TRBC1−	2.17
T‐17	T‐LGL	PB	CD3+, CD8+, CD2+, CD5+, CD7dim+, TRBC1−	48.93
T‐18	T‐LGL	PB	CD3+, CD8+, CD2+, TRBC1−	75.3
T‐19	T‐LGL	PB	CD3+, CD8+, CD2+, CD5+, CD7+, TRBC1−	51.47
T‐20	T‐LGL	BM	CD3+, CD8+, CD2+, TRBC1−	1.54

Abbreviations: AF, ascitic fluid; ALCL, anaplastic large‐cell lymphoma; BM, bone marrow; CSF, cerebrospinal fluid; ENKTCL, extranodal NK/T‐cell lymphoma; PB, peripheral blood; PF, pleural fluid; PTCL, peripheral T‐cell lymphoma; sEBV + TCL, systemic EBV + T‐cell lymphoma of childhood; TCL, T‐cell lymphoma; TFH, follicular helper T‐cell; TLGL, T‐cell large granulocyte lymphocytic leukemia; T‐PLL, T‐cell prolymphocytic leukemia.

^a^ Among the markers CD4, CD8, CD2, CD5, CD7, and CD56, those not specified were negative.

Notably, among cases with < 5% clonal T cells, T‐7 (systemic EBV+ T‐cell lymphoma, 0.45%) and T‐20 (T‐LGL, 1.54%) were follow‐up bone marrow specimens from confirmed T‐cell lymphomas in bone marrow, while T‐15 and T‐16 (follicular helper T‐cell lymphoma, NOS, 4.90% and 2.17%, respectively) were peripheral blood specimens from patients with prior bone marrow diagnoses, with flow cytometric findings consistent with prior immunophenotypic findings.

### 3.4 T‐Cell Clones of Uncertain Significance

**FIGURE 3:**
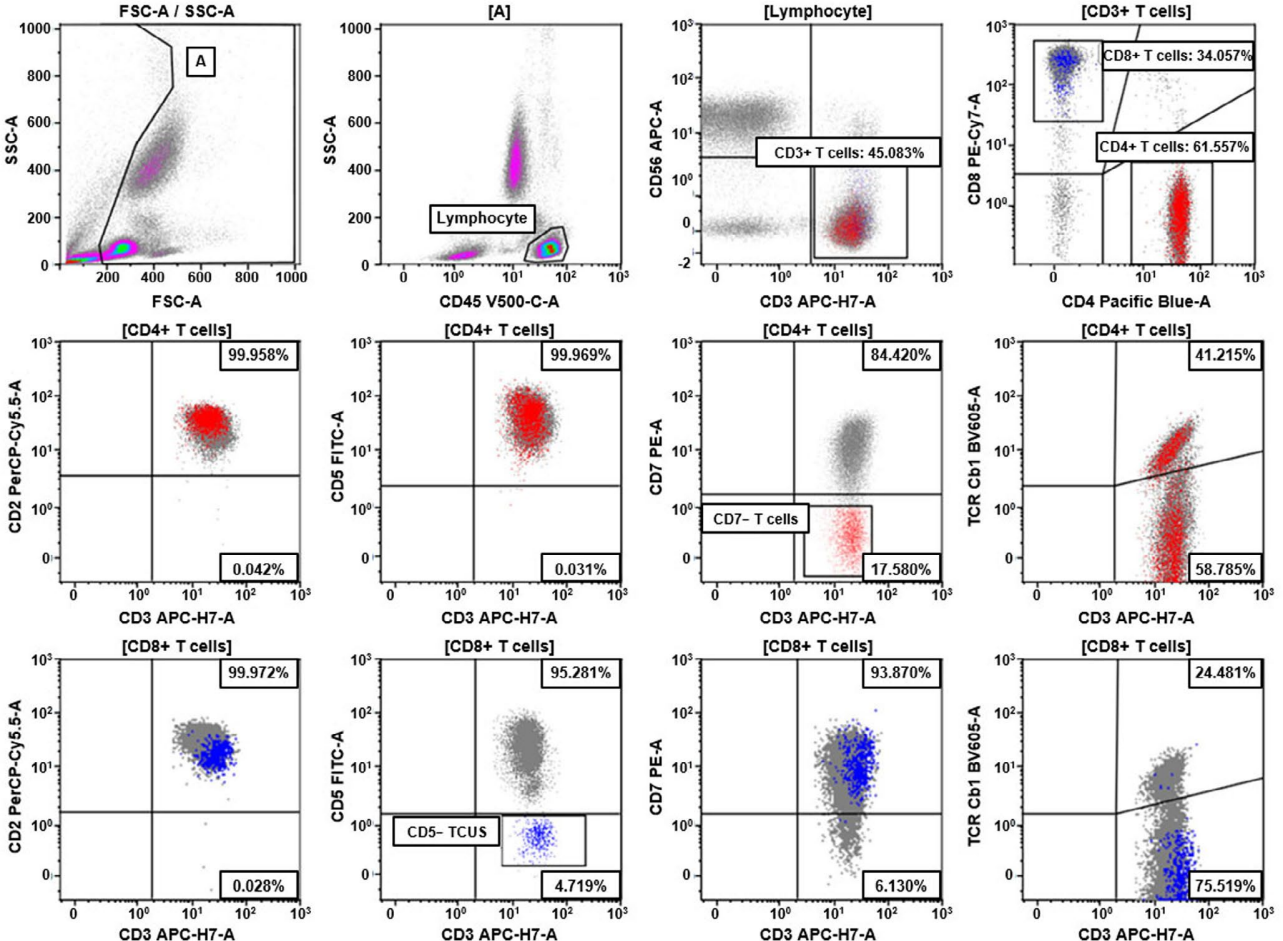
Flow cytometric analysis of TRBC1 expression in a patient (U‐6) with idiopathic cytopenia of undetermined significance. Both total CD4+ and CD8+ T cells exhibit polytypic TRBC1 expression. A subset of CD4+ T cells with CD7 loss (red dots) shows polytypic TRBC1 expression. In contrast, a subset of CD8+ T cells with CD5 loss (blue dots) demonstrates a distinctly monotypic TRBC1– pattern (TRBC1–, 99.0%; TRBC1+, 1.0%), which was markedly outside the range observed in the CD3+CD7+CD5– T‐cell population from non‐T‐cell lymphoma bone marrow aspirates. This highly skewed TRBC1 expression pattern, combined with the immunophenotypic aberrancy, supports the interpretation of T‐cell clonality of undetermined significance (TCUS) in the absence of other evidence of T‐cell lymphoma.

TCUS was detected in 21 (12.7%) of 166 non‐T‐cell lymphoma infiltration specimens (Table 3). Of these, 10 showed monotypic TRBC1+ expression, and 11 showed monotypic TRBC1− expression. In bone marrow aspirates, 18 TCUS cases were identified: five (27.8%) in CD4+ T cells and 13 (72.2%) in CD8+ T cells. A representative plot of TCUS is presented in Figure 3. The median proportion of clonal T cells in TCUS was 0.35% (range, 0.04%–3.99%), which was significantly lower than in T‐cell lymphomas (median 13.65%, *p <* 0.001). These cases were predominantly observed in patients with cytopenic conditions including aplastic anemia and pure red cell aplasia (*n* = 9), and hemophagocytic lymphohistiocytosis (*n* = 6). Among the six hemophagocytic lymphohistiocytosis‐associated TCUS cases, circulating EBV DNA was positive in three (U‐7, U‐11, and U‐14), negative in one (U‐12), and not assessed in two (U‐3 and U‐10). Additionally, three TCUS cases were detected in body fluids from patients with neurological conditions (U‐20) and underlying lymphomas (U‐15 and U‐17). Notably, in four patients (U‐10, U‐17, U‐19, U‐21) previously diagnosed with T‐cell lymphoma, T‐cell subpopulations were observed in bone marrow or pleural fluid specimens at extremely low frequencies (0.04%–0.81%). These subpopulations were classified as TCUS not only due to their minimal clone size but also because they either did not align with histologic findings or lacked additional clinical evidence of lymphoma infiltration in the respective specimens.

**TABLE 3:** Clinical and immunophenotypic characteristics of cases with T‐cell clones of uncertain significance (*n* = 21).

Case no.	Clinical information	Specimen	Immunophenotype^a^	Proportion (%)
U‐1	PRCA	BM	CD3+, CD8+, CD2+, CD7+, TRBC1−	3.99
U‐2	Anemia, NOS	BM	CD3+, CD56dim+, CD8dim+, CD2+, CD5+, CD7+, TRBC1−	0.18
U‐3	HLH	BM	CD3+, CD56+, CD2+, CD7+, TRBC1−	0.64
U‐4	AA	BM	CD3+, CD8+, CD2+, CD7+, TRBC1−	0.18
U‐5	CCUS	BM	CD3+, CD4+, TRBC1−	0.30
U‐6	ICUS	BM	CD3+, CD8+, CD2+, CD7+, TRBC1−	0.36
U‐7	BM involvement of DLBCL + HLH	BM	CD3+, CD8+, CD2+, CD7+, TRBC1+	0.11
U‐8	AA	BM	(P1) CD3+, CD2+, CD7+, TRBC1−; (P2) CD3+, CD4+, CD2+, CD7+, TRBC1−	(P1) 0.41; (P2) 0.10
U‐9	Anemia, NOS	BM	CD3+, CD8+, CD2+, CD7+, TRBC1−	0.12
U‐10	BM involvement of NKTCL + HLH	BM	CD3+, CD4+, TRBC1−^b^	0.81
U‐11	HLH	BM	CD3+, CD8+, CD2+, CD7+, TRBC1−	0.39
U‐12	HLH	BM	(P1) CD3+, CD4+, CD2+, CD5+, TRBC1−; (P2) CD3+, CD8+, CD2+, CD7+, TRBC1−	(P1) 0.33; (P2) 0.11
U‐13	PRCA	BM	(P1) CD3++, CD8+, CD5dim+, CD2+, CD7+, TRBC1−; (P2) CD3+, CD8+, CD2+, CD7+, TRBC1−	(P1) 1.12; (P2) 0.21
U‐14	BM involve of DLBCL + HLH	BM	CD3+, CD56+, CD8+, CD2+, CD7+, TRBC1−	0.26
U‐15	B‐cell neurolymphomatosis	CSF	CD3+, CD8++, CD2+, CD5+, CD7+, TRBC1−	3.76^d^
U‐16	PRCA	BM	CD3+, CD8+, CD2+, CD7+, TRBC1−	3.03
U‐17	THF cell lymphoma	PF	CD3+, CD4+, TRBC1−^c^	0.07
U‐18	Fever, vasculitis	BM	CD3+, CD56+, CD8+, CD2+, CD7+, TRBC1+	0.40
U‐19	ENKTCL	BM	CD3+, CD8+, CD2+, CD5+, TRBC1−^c^	0.28
U‐20	CIDP	CSF	CD3+, CD8+, CD2+, CD5+, TRBC1−	10.27^d^
U‐21	THF cell lymphoma	BM	CD3+, CD8+, CD2+, CD7+, TRBC1−^c^	0.04

Abbreviations: AA, aplastic anemia; BM, bone marrow; CCUS, clonal cytopenia of unknown significance; CIDP, chronic inflammatory demyelinating polyneuropathy; CSF, cerebrospinal fluid; DLBCL, diffuse large B‐cell lymphoma; ENKTCL, extranodal NK/T‐cell lymphoma; HLH, hemophagocytic lymphohistiocytosis; PF, pleural fluid; PRCA, pure red cell aplasia; TFH, follicular help T‐cell.

^a^ Among the markers CD4, CD8, CD2, CD5, and CD7, those not specified were negative.

^b^ An abnormal NK cell population (CD3−, CD56+, CD2+, CD5−, and CD7−) was simultaneously detected by flow cytometry, with histological findings concordant with these immunophenotypic characteristics. Given these observations, the detected T‐cell subclone was classified as TCUS.

^c^ This case was previously diagnosed with a specific T‐cell lymphoma in different tissues. Flow cytometry was performed on each specimen, but no additional clinical evidence of lymphoma infiltration was found. Therefore, the findings were classified as TCUS.

^d^ The proportion of the population was overestimated due to a low white blood cell count.

## 4 Discussion

This study provides a comprehensive evaluation of TRBC1 flow cytometry for T‐cell lymphoma diagnosis across various clinical specimens, providing several critical insights into TRBC1 expression patterns, diagnostic potential, and the limitations associated with TCUS.

Previous studies have reported TRBC1 expression in healthy peripheral blood, showing a slightly higher expression in CD4+ T cells compared to CD8+ T cells. Munoz‐Garcia et al. reported mean TRBC+ expression of 43% and 35% in CD4+ and CD8+ T cells in normal and reactive peripheral blood, respectively (mean TRBC1+/TRBC1− ratios, 0.75 and 0.53, respectively), with 99.7% reference intervals of 24%–62% and 8.3%–61% [16]. Similarly, Castillo et al. reported mean TRBC+ expression of 42% and 38% (mean TRBC1+/TRBC1− ratios: 0.72 and 0.61), with 99.7% reference intervals of 29.4%–54.6% and 23.9%–52.1% in healthy peripheral blood [17]. Berg et al. reported comparable findings in non‐T‐cell lymphoma specimens, with median values of 43.8% and 37.9% for CD4+ and CD8+ T cells, respectively, and 95th percentile ranges of 35.8%–51.1% and 36.5%–50.8% [13]. While we observed similar patterns of TRBC1 expression to previous studies, we found relatively lower TRBC1 expression (median 26.3%) in CD8+ T cells from healthy peripheral blood. In non‐T‐cell lymphoma bone marrow specimens, TRBC1 expression in T cells was slightly elevated compared to that in peripheral blood from healthy individuals. Given that TRBC1 expression patterns in reactive bone marrow specimens were similar to those in the overall non‐T‐cell lymphoma bone marrow specimens, this subtle increase in TRBC1 expression is likely attributable to tissue‐specific immunologic characteristics, rather than reactive or disease‐associated changes. These findings may suggest the potential benefit of establishing specimen‐specific reference ranges across different clinical settings.

From a diagnostic perspective, several studies have shown the utility of TRBC1 as a clonal marker in various T‐cell lymphomas, particularly in T‐LGL where clonal T cells are readily accessible in peripheral blood for flow cytometric analysis [4, 13, 16, 17, 18, 19]. Our study confirmed the diagnostic utility of TRBC1 across various T‐cell lymphomas, including anaplastic large‐cell lymphoma, extranodal NK/T‐cell lymphoma, peripheral T‐cell lymphoma, T‐cell prolymphocytic leukemia, follicular helper T‐cell lymphoma, and T‐LGL, while notably showing a predominance (65%) of TRBC1− (potentially TRBC2+) cases, which may have significant implications for predicting patient populations suitable for emerging TRBC1 and TRBC2 targeted therapies [20, 21]. Meanwhile, the gold standard for diagnosing T‐cell lymphoma in this study was histopathologic confirmation, primarily through bone marrow or tissue biopsy, supplemented by clinical correlation. Most T‐cell lymphoma cases in our study were diagnosed by this standard prior to or concurrently with flow cytometry analysis using TRBC1. Among the 20 T‐cell lymphoma cases, four were classified based on monotypic TRBC1 expression without antigen aberrancies. In three cases (T‐1, T‐4, and T‐9), the diagnosis was supported by histopathologic and clinical findings. One T‐LGL case (T‐19) was diagnosed based on large granular lymphocytosis and monotypic TRBC1− CD8+ T cells in peripheral blood, without definitive histological confirmation. The diagnosis was further supported by these cells comprising approximately half of total leukocytes and the majority of peripheral blood lymphocytes. However, diagnosing lymphoma based solely on peripheral blood flow cytometry requires careful interpretation.

Clinical implementation of TRBC1 flow cytometry has led to frequent detection of small subsets of TCUS in patients without clinical evidence of T‐cell lymphoma, with proposed cutoff values of 5%–20% of lymphocytes and 0.05–0.5 × 10^9^/L [10, 12]. The prevalence of TCUS has been reported to range from 16% to 26% [10, 12], though these rates may vary depending on the methodology and study population [22]. In our study, we observed a frequency of 12.7%, with predominance in CD8+ T cells. This CD8+ predominance, also noted in a previous study [10], likely reflects clonal expansion in immune responses, as CD8+ T cells can persist in the memory pool after antigenic exposure, whereas, CD4+ T cells are more tightly constrained and rarely reach detection thresholds [23].

In our study, half of TCUS cases detected in bone marrow were associated with cytopenia, likely due to associations between clonal T cells and various cytopenic conditions [24, 25]. For instance, aplastic anemia is known to be mediated by aberrant T‐cell immune responses, leading to bone marrow hematopoietic dysfunction and peripheral blood pancytopenia [24]. Similarly, acquired pure red cell aplasia is attributed to dysregulated T‐cell‐mediated immunity, with clonal CD8+ T cells potentially involved in selective inhibition of erythroid progenitors [25]. Clonal T‐cell populations are also frequently observed in myelodysplastic neoplasms, likely reflecting a reactive phenomenon [25]. Additionally, one‐third of bone marrow TCUS cases were associated with hemophagocytic lymphohistiocytosis, with EBV DNA detected in three cases supporting previous findings of a relation between aberrant clonal T‐cell populations and EBV‐positive hemophagocytic lymphohistiocytosis [11]. Therefore, the presence of clonal T cells in these clinical contexts should not be interpreted in isolation as indicative of T‐cell lymphoma. Furthermore, our findings suggest that TRBC1 flow cytometry has limited utility in detecting minimal involvement of T‐cell lymphoma, as the possibility of TCUS cannot be definitively excluded. In TCUS cases occurring in patients with a history of T‐cell lymphoma, interpretation relied on comparison with prior immunophenotypic profiles and current clinical findings to exclude minimal disease involvement. However, in scenarios where the lymphoma undergoes immunophenotypic shift, distinguishing TCUS from true residual lymphoma becomes particularly challenging. These considerations underscore the importance of interpreting small clonal T‐cell populations with a comprehensive clinical and pathological context.

Our study has several limitations. First, as a retrospective study, there is potential for selection bias due to the selective testing of certain clinical patients, with unevenly distributed sample sizes across different groups and only a small number of T‐cell lymphoma patients included in the analysis. Second, when performing TRBC1 flow cytometry, it is important to note that the TRBC1 assay is specifically applicable to αβ T cells, with limitations in assessing cells lacking surface TRBC1 or TRBC2 expression, such as γδ T cells, benign thymocytes, and surface CD3− lymphomas [13]. Two approaches can address this specificity: direct selection of αβ T cells using specific antibodies [22] or exclusion of γδ T cells based on their characteristic expression patterns as CD4−/CD8− or CD8dim T cells [15]. While we employed the latter approach in our study, there may have been potential limitations in accurate γδ T‐cell discrimination. Third, the typically small size of gated populations made it challenging to obtain reliable data on TRBC1 expression in various minor T‐cell subpopulations. Finally, as noted above, in specimens previously diagnosed with T‐cell lymphoma, low‐frequency clonal populations lacking histologic or clinical evidence of lymphoma and discordant with prior immunophenotypes were classified as TCUS, but the possibility of minimal residual disease could not be entirely excluded.

In conclusion, this study demonstrated the clinical utility of flow cytometric TRBC1 assessment in diagnosing T‐cell lymphomas. By analyzing a large cohort of specimens without T‐cell lymphoma involvement, we characterized baseline TRBC1 expression patterns across diverse specimen types and identified TCUS across various clinical settings. In particular, by presenting TRBC1 flow cytometry results from real‐world clinical practice, this study contributes to improving the clinical interpretability of TRBC1 findings. These results underscore the importance of interpreting TRBC1 flow cytometry results within the appropriate clinical context. Furthermore, exploring the combination of TRBC1 flow cytometry with other diagnostic tools may enhance the specificity and sensitivity of T‐cell lymphoma diagnosis.

## Author Contributions

S.Y. and H.‐Y.K. designed the study. S.Y., B.K., and H.‐Y.K. analyzed the data. S.E.Y. treated patients. S.Y. and H.‐Y.K. wrote and edited the manuscript. H.‐J.K. reviewed the manuscript. All authors reviewed and approved the final version of the manuscript.

## Ethics Statement

This study was approved by the Institutional Review Board of Samsung Medical Center, Seoul, Korea (2024‐09‐004).

## Consent

This study was conducted with a waiver of informed consent due to its retrospective design.

## Conflicts of Interest

The authors declare no conflicts of interest.

## Supporting information


Table S1.

## Data Availability

The data that support the findings of this study are available from the corresponding author upon reasonable request.
