# Supplementary material for: Flow Cytometric Assessment of TRBC1 Expression for the Diagnosis of T‐Cell Lymphoma: Clinical Utility and Pitfalls Related to T‐Cell Clones of Uncertain Significance
Source: Int J Lab Hematol. 2025 Jun 16;47(5):840–8. doi: 10.1111/ijlh.14508 (PMC12426804; doi:10.1111/ijlh.14508)
Supplement: Supplementary file 1 — Table S1. [file IJLH-47-840-s001.pdf]

Table S1. Clinical information of 166 specimens without T-cell lymphoma involvement. Total of 109 bone marrow, 8 peripheral blood, and 49 body fluid specimens were analyzed by flow cytometry.

Case No.	Specimen	Clinical information
N-1	Bone marrow	Classic Hodgkin lymphoma
N-2	Bone marrow	Fever of unknown origin
N-3	Bone marrow	Hemophagocytic lymphohistiocytosis
N-4	Peripheral blood	Fever of unknown origin
N-5	Bone marrow	Not available
U-1	Bone marrow	Pure red cell aplasia
N-6	Bone marrow	TFH cell lymphoma, NOS
N-7	Pleural fluid	Primary mediastinal large B cell lymphoma
N-8	Bone marrow	Peripheral T cell lymphoma
N-9	Pericardial fluid	Primary mediastinal large B cell lymphoma
N-10	Bone marrow	Peripheral T cell lymphoma
N-11	Bone marrow	Diffuse large B-cell lymphoma, Activated B-cell Type
N-12	Cerebrospinal fluid	Multiple sclerosis
N-13	Bone marrow	Immune thrombocytopenia
N-14	Bone marrow	Not available
U-2	Bone marrow	Anemia, NOS
N-15	Bone marrow	Monomorphic Epitheliotropic Intestinal T cell lymphoma
N-16	Bone marrow	Lymphadenopathy
U-3	Bone marrow	Hemophagocytic lymphohistiocytosis
N-17	Cerebrospinal fluid	Primary diffuse large B-cell lymphoma of the central nervous system
N-18	Bone marrow	Hepatosplenic mature T-cell lymphoma
N-19	Bone marrow	Diffuse large B-cell lymphoma, Activated B-cell Type
N-20	Bone marrow	Adult-onset Still's disease
U-4	Bone marrow	Aplastic anemia
N-21	Bone marrow	Kikuchi's disease of neck
N-22	Bone marrow	Peripheral T cell lymphoma
N-23	Cerebrospinal fluid	Rapidly progressive dementia
N-24	Bone marrow	TFH cell lymphoma, NOS
N-25	Bone marrow	Aplastic anemia

U-5	Bone marrow	Clonal cytopenia of undetermined significance
U-6	Bone marrow	Idiopathic cytopenia of undetermined significance
N-26	Pleural fluid	B-cell acute lymphoblastic leukemia
N-27	Pleural fluid	Chylothorax
N-28	Bone marrow	Plasma cell myeloma
N-29	Cerebrospinal fluid	Extranodal NK/T-cell lymphoma
U-7	Bone marrow	Diffuse large B-cell lymphoma, Hemophagocytic lymphohistiocytosis
N-30	Bone marrow	Anaplastic large-cell lymphoma, ALK positive
N-31	Bone marrow	Diamond-Blackfan anemia
U-8	Bone marrow	Aplastic anemia
N-32	Bone marrow	Classic Hodgkin lymphoma
N-33	Bone marrow	Papillary thyroid carcinoma
U-9	Bone marrow	Anemia, NOS
N-34	Bone marrow	Nodal Peripheral T cell lymphoma
N-35	Peripheral blood	Kidney transplantation status
N-36	Peripheral blood	Anaplastic large-cell lymphoma, ALK positive
N-37	Cerebrospinal fluid	Primary diffuse large B-cell lymphoma of central nervous system
N-38	Bone marrow	Hemophagocytic lymphohistiocytosis
N-39	Bone marrow	B-cell acute lymphoblastic leukemia
N-40	Bone marrow	TFH cell lymphoma, angioimmunoblastic type
N-41	Bone marrow	Chronic active Epstein-Barr virus infection
N-42	Bone marrow	Fever of unknown origin
N-43	Bone marrow	Diffuse large B-cell lymphoma, Activated B-cell Type
N-44	Cerebrospinal fluid	Primary diffuse large B-cell lymphoma of central nervous system
N-45	Cerebrospinal fluid	Multiple sclerosis
N-46	Bone marrow	Anaplastic large-cell lymphoma, ALK positive
N-47	Bone marrow	Aplastic anemia
N-48	Cerebrospinal fluid	Autoimmune encephalitis
U-10	Bone marrow	NK/T-cell lymphoma, Hemophagocytic lymphohistiocytosis
N-49	Bone marrow	Diffuse large B-cell lymphoma, Activated B-cell Type
N-50	Bone marrow	Not available

U-11	Bone marrow	Hemophagocytic lymphohistiocytosis
N-51	Pericardial fluid	Primary mediastinal large B-cell lymphoma
N-52	Bone marrow	Fever of unknown origin
N-53	Peripheral blood	Chronic lymphocytic leukemia
U-12	Peripheral blood	Hemophagocytic lymphohistiocytosis
N-54	Bone marrow	Follicular T-cell lymphoma
N-55	Bone marrow	Acute hepatitis
N-56	Bone marrow	Leukocytosis
N-57	Peripheral blood	Miliary tuberculosis
U-13	Bone marrow	Pure red cell aplasia
N-58	Bone marrow	Hemophagocytic lymphohistiocytosis
N-59	Bone marrow	Extranodal NK/T-cell lymphoma
N-60	Bone marrow	Myelodysplastic syndrome
N-61	Bone marrow	Diffuse large B-cell lymphoma, Activated B-cell Type
N-62	Bone marrow	Follicular T-cell lymphoma
N-63	Peripheral blood	TFH cell lymphoma, angioimmunoblastic type
N-64	Bone marrow	Extranodal NK/T-cell lymphoma
N-65	Pleural fluid	TFH cell lymphoma, angioimmunoblastic type
N-66	Bone marrow	Classic Hodgkin lymphoma
U-14	Ascitic fluid	Diffuse large B-cell lymphoma, Hemophagocytic lymphohistiocytosis
N-67	Cerebrospinal fluid	Multiple sclerosis
N-68	Bone marrow	Extranodal NK/T-cell lymphoma
N-69	Cerebrospinal fluid	Primary diffuse large B-cell lymphoma of central nervous system
N-70	Cerebrospinal fluid	Multiple sclerosis
N-71	Cerebrospinal fluid	Multiple myeloma
U-15	Bone marrow	B-cell neurolymphomatosis
U-16	Pleural fluid	Pure red cell aplasia
N-72	Bone marrow	Peripheral T cell lymphoma
N-73	Bone marrow	TFH cell lymphoma, angioimmunoblastic type
N-74	Bone marrow	Pancytopenia
N-75	Bone marrow	Fever of unknown origin
N-76	Cerebrospinal fluid	Chronic inflammatory demyelinating polyneuropathy
N-77	Cerebrospinal fluid	Left hypoglossal nerve palsy

N-78	Bone marrow	Vascular dementia
N-79	Bone marrow	Monomorphic Epitheliotropic Intestinal T cell lymphoma
U-17	Bone marrow	TFH cell lymphoma, NOS
N-80	Bone marrow	Fever of unknown origin
N-81	Cerebrospinal fluid	Post-zoster neuralgia
N-82	Bone marrow	Fever of unknown origin
N-83	Bone marrow	Fever of unknown origin
N-84	Bone marrow	Anaplastic large-cell lymphoma, ALK negative
N-85	Bone marrow	Noonan's syndrome
N-86	Cerebrospinal fluid	Chronic inflammatory demyelinating polyneuropathy
N-87	Bone marrow	Aggressive NK cell leukemia
N-88	Bone marrow	Hepatosplenic T-cell Lymphoma
N-89	Cerebrospinal fluid	Neurolymphomatosis
N-90	Cerebrospinal fluid	Brachial plexopathy
N-91	Cerebrospinal fluid	Central nervous system vasculitis
N-92	Cerebrospinal fluid	Central-type facial palsy
N-93	Bone marrow	Fever of unknown origin
N-94	Cerebrospinal fluid	Diplopia
U-18	Bone marrow	Fever, vasculitis
N-95	Peripheral blood	Acute myeloid leukemia
N-96	Bone marrow	Monomorphic Epitheliotropic Intestinal T-cell Lymphoma
N-97	Bone marrow	Myelodysplastic syndrome
N-98	Bone marrow	Fever of unknown origin
N-99	Cerebrospinal fluid	Guillain-Barre syndrome
U-19	Cerebrospinal fluid	Extranodal NK/T-cell lymphoma
N-100	Pericardial fluid	Mediastinitis
N-101	Bone marrow	T-cell lymphoblastic lymphoma
N-102	Bone marrow	Extranodal NK/T-cell lymphoma
N-103	Cerebrospinal fluid	Dementia
N-104	Cerebrospinal fluid	Myelopathy
N-105	Cerebrospinal fluid	Central nervous system vasculitis
N-106	Cerebrospinal fluid	Central nervous system vasculitis
N-107	Cerebrospinal fluid	Facial nerve palsy

N-108	Bone marrow	Aggressive NKcell Leukemia
N-109	Bone marrow	Intestinal T-cell lymphoma
N-110	Bone marrow	Kikuchi's disease
N-111	Bone marrow	Kikuchi's disease
N-112	Bone marrow	Systemic EBV+ T-cell lymphoma of Childhood
N-113	Cerebrospinal fluid	Cerebral infarction
N-114	Cerebrospinal fluid	Cerebral infarction
N-115	Bone marrow	Acute myeloid leukemia
N-116	Bone marrow	Diffuse large B-cell lymphoma, Germinal center B-cell type
N-117	Cerebrospinal fluid	Myelitis
N-118	Cerebrospinal fluid	Diffuse large B-cell lymphoma, Activated B-cell Type
N-119	Bone marrow	Diffuse large B-cell lymphoma, Activated B-cell Type
N-120	Bone marrow	Fever of unknown origin
N-121	Bone marrow	Peripheral T-cell lymphoma
N-122	Cerebrospinal fluid	Guillain-Barre syndrome
U-20	Bone marrow	Chronic inflammatory demyelinating polyneuropathy
N-123	Cerebrospinal fluid	Autoimmune encephalitis
N-124	Cerebrospinal fluid	Cerebral infarction
N-125	Bone marrow	Diffuse large B-cell lymphoma, Germinal center B-cell type
N-126	Bone marrow	Fever of unknown origin
N-127	Cerebrospinal fluid	Demyelinating disease of central nervous system
N-128	Bone marrow	Hemophagocytic lymphohistiocytosis
N-129	Bone marrow	Anaplastic large-cell lymphoma, ALK positive
N-130	Bone marrow	TFH cell lymphoma, angioimmunoblastic type
N-131	Bone marrow	T-cell lymphoblastic lymphoma
N-132	Cerebrospinal fluid	Diffuse glioneuronal tumor
N-133	Bone marrow	Adult-onset Still's disease
U-21	Bone marrow	TFH cell lymphoma, NOS
N-134	Bone marrow	Peripheral T-cell lymphoma
N-135	Cerebrospinal fluid	Headache
N-136	Bone marrow	Anemia, NOS
N-137	Bone marrow	Anemia, NOS
N-138	Bone marrow	Neutropenia

N-139	Bone marrow	Peripheral T cell lymphoma
N-140	Bone marrow	To rule out mesenteric lymphoma
N-141	Pleural fluid	Peripheral T-cell lymphoma
N-142	Bone marrow	Fever of unknown origin
N-143	Bone marrow	Extranodal NK/T-cell lymphoma
N-144	Bone marrow	To rule out lymphoma
N-145	Cerebrospinal fluid	Vasculitic polyneuropathy

Abbreviations: NK, natural killer T-cell; TFH, follicular helper T-cell
